# Supplementary material for: Targeted isolation of Methanobrevibacter strains from fecal samples expands the cultivated human archaeome
Source: Nat Commun. 2024 Aug 31;15:7593. doi: 10.1038/s41467-024-52037-7 (PMC11366006; doi:10.1038/s41467-024-52037-7)
Supplement: Supplementary file 1 — Supplementary Information [file 41467_2024_52037_MOESM1_ESM.pdf]

Targeted isolation of *Methanobrevibacter* strains from fecal samples expands the cultivated human archaeome

➲ Supplementary Information

Stefanie Duller[1], Simone Vrbancic[1], Łukasz Szydłowski[2,3], Alexander Mahnert[1,4], Marcus Blohs[1], Michael Predl[5,6], Christina Kumpitsch[1,2], Verena Zrim[7], Christoph Högenauer[8], Tomasz Kosciolek[2,3,9], Ruth A. Schmitz[10], Anna Eberhard[1], Melanie Dragovan[1], Laura Schmidberger[1], Tamara Zurabischvili[1], Viktoria Weinberger[1], Adrian Mathias Moser[8], Dagmar Kolb[11], Dominique Pernitsch[11], Rokhsareh Mohammadzadeh[1], Torben Kühnast[1], Thomas Rattei[5], Christine Moissl-Eichinger[1,4*]

[1] D&R Institute of Hygiene, Microbiology and Environmental Medicine, Medical University of Graz, Graz, Austria;

[2] Malopolska Centre of Biotechnology, Jagiellonian University in Krakow, Poland;

[3] Sano Centre for Computational Medicine, Krakow, Poland

[4] BioTechMed Graz, Graz, Austria;

[5] Centre for Microbiology and Environmental Systems Science, University of Vienna, Vienna, Austria;

[6] Doctoral School Microbiology and Environmental Science, University of Vienna, Vienna, Austria

[7] Center for Medical Research, Medical University of Graz, Graz, Austria;

[8] Division of Gastroenterology and Hepatology, Department of Internal Medicine, Medical University of Graz, Graz, Austria;

[9] Department of Data Science and Engineering, Silesian University of Technology, Gliwice, Poland;

[10] Institute for General Microbiology, Christian Albrechts University, Kiel, Germany;

[11] Core Facility Ultrastructure Analysis, Medical University of Graz, Graz, Austria;

*Corresponding author

christine.moissl-eichinger@medunigraz.at

Supplementary Figures

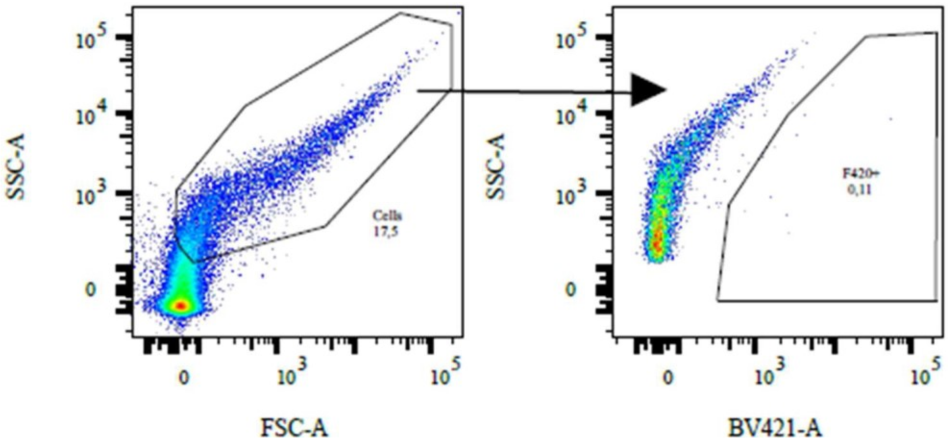

Supplementary Figure S1: **General gating strategy of FACS sorting.** Negative control shown on the left. The F420+ gate in the second dot plot represents the sort gate.

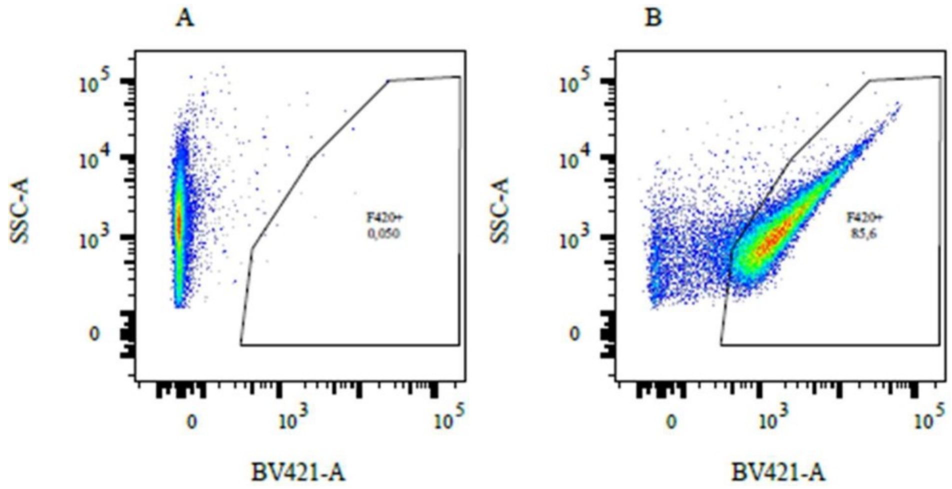

Supplementary Figure S2: **FACS sorting: Example of a (A)** CH_4**-negative and (B)** CH_4**-positive culture.**

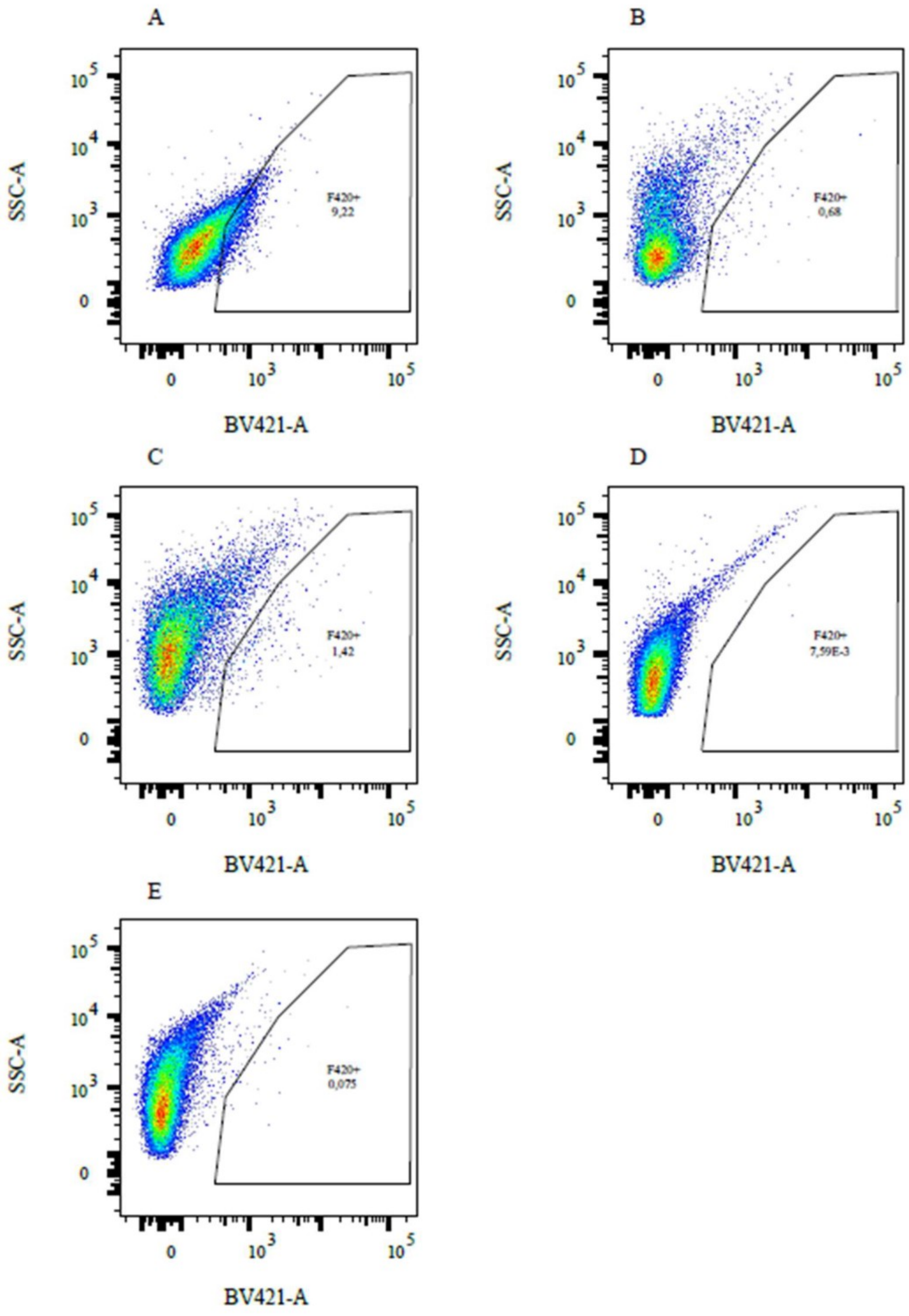

Supplementary Figure S3: FACS Sorting of pure cultures. (A) *Methanobrevibacter smithii*, (B) *Methanosphaera stadtmanae*, (C) *Methanomassiliicoccus luminyensis*, (D) *Bacteroides thetaiotaomicron*, (E) *Christensenella minuta.*

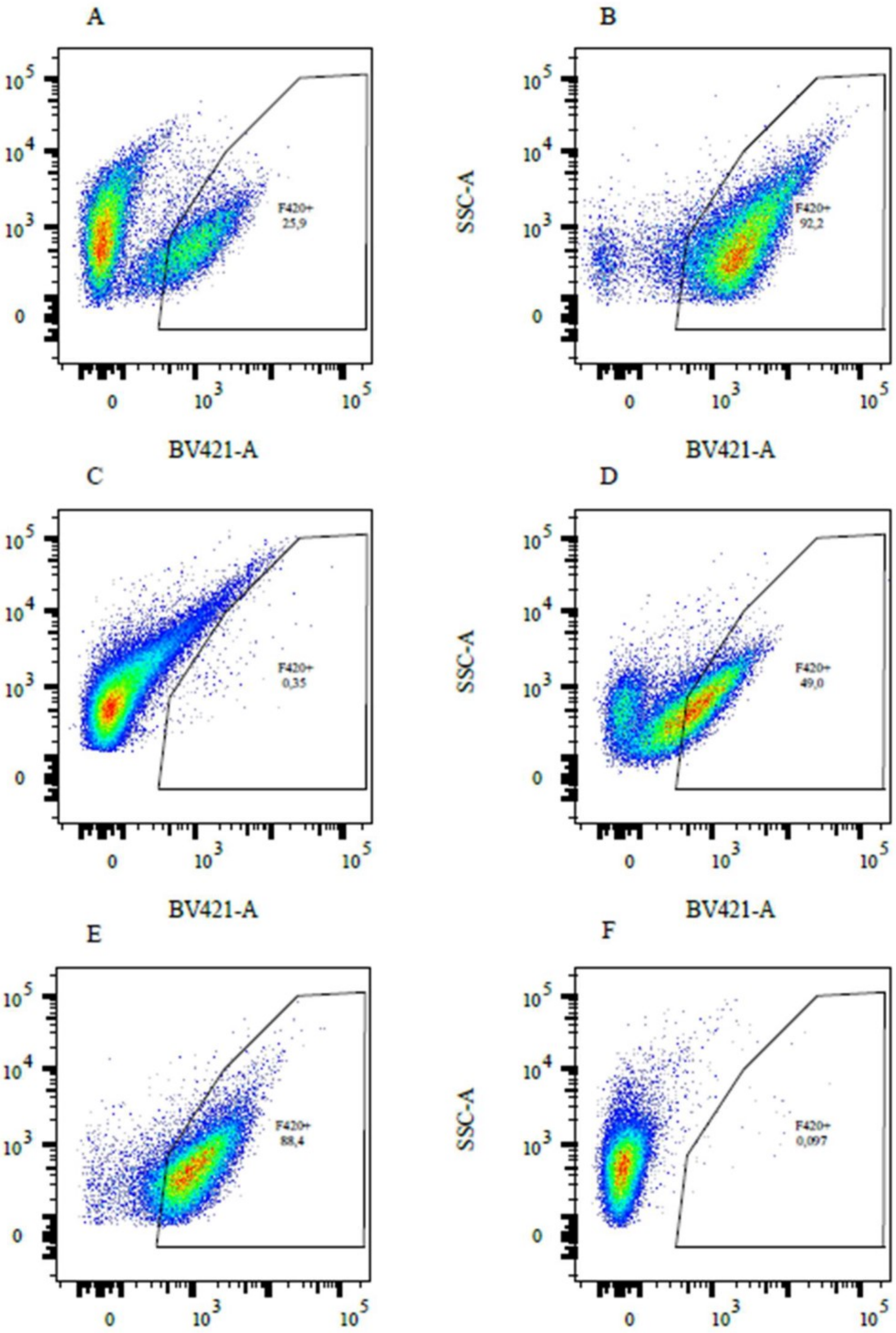

Supplementary Figure S4: FACS Sorting of mixed cultures. (A) *Christensenella minuta + Methanosphaera stadtmanae*, (B) *Christensenella minuta + Methanobrevibacter smithii*, (C) *Christensenella minuta + Methanomassiliicoccus luminyensis*, (D) *Bacteroides thetaiotaomicron + Methanosphaera stadtmanae*, (E) *Bacteroides thetaiotaomicron + Methanobrevibacter smithii*, (F) *Bacteroides thetaiotaomicron + Methanomassiliicoccus luminyensis***.**

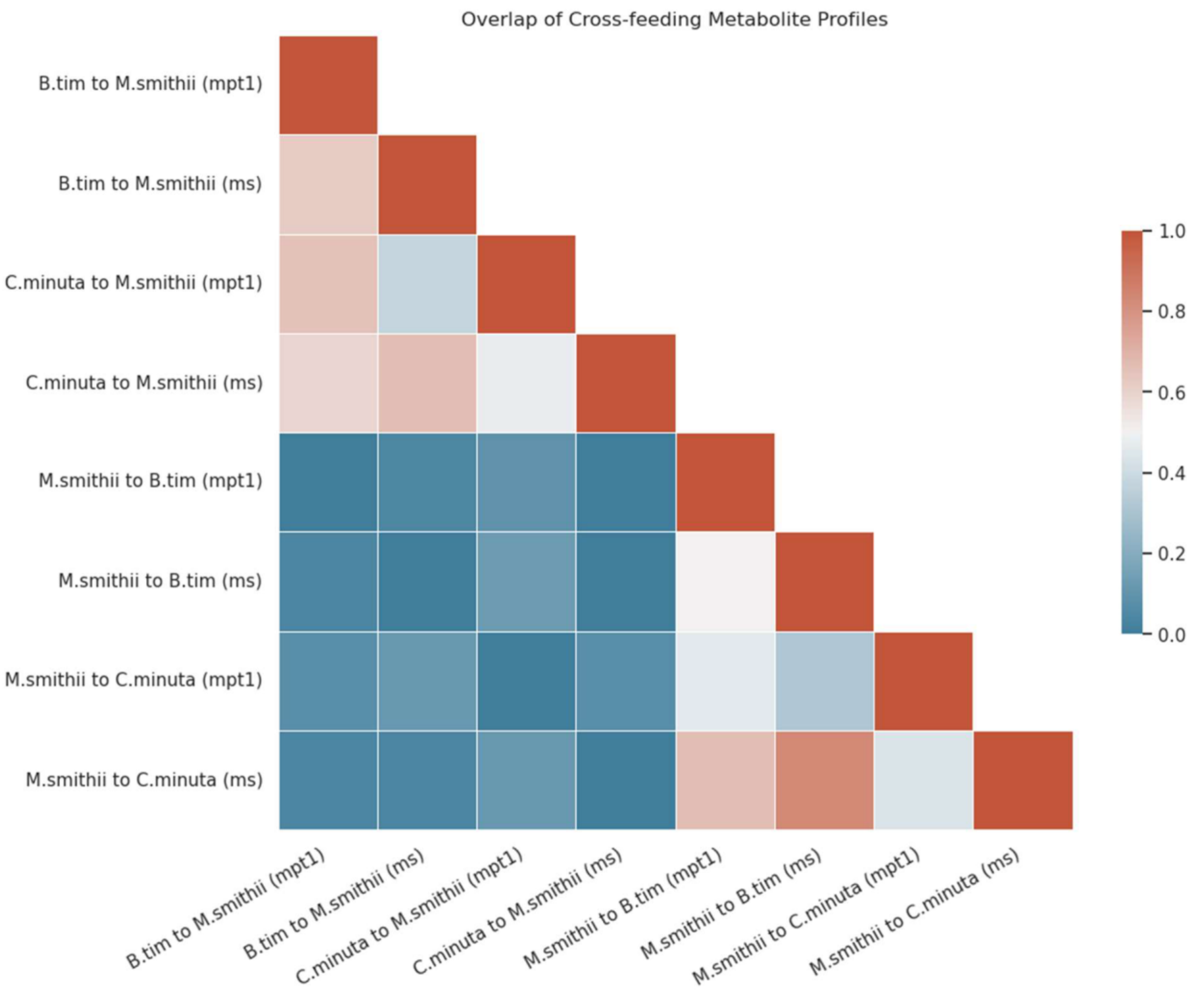

Supplementary Figure S5: Comparison of cross-feeding interaction profiles in simulated co-cultures. Interaction profiles consist of all metabolites produced by organism A and consumed by organism B in a co-culture of A and B. Overlap is calculated as the intersection of cross-fed metabolites divided by the union of metabolites of cross-fed metabolites. Across all co-cultures, interaction profiles of the same direction between domains (bacteria to *M. smithii*, or *M. smithii* to bacteria) have higher overlap, than interaction profiles with different directions. Underlying information is available in our GitHub repository (Duller et al., 2024).

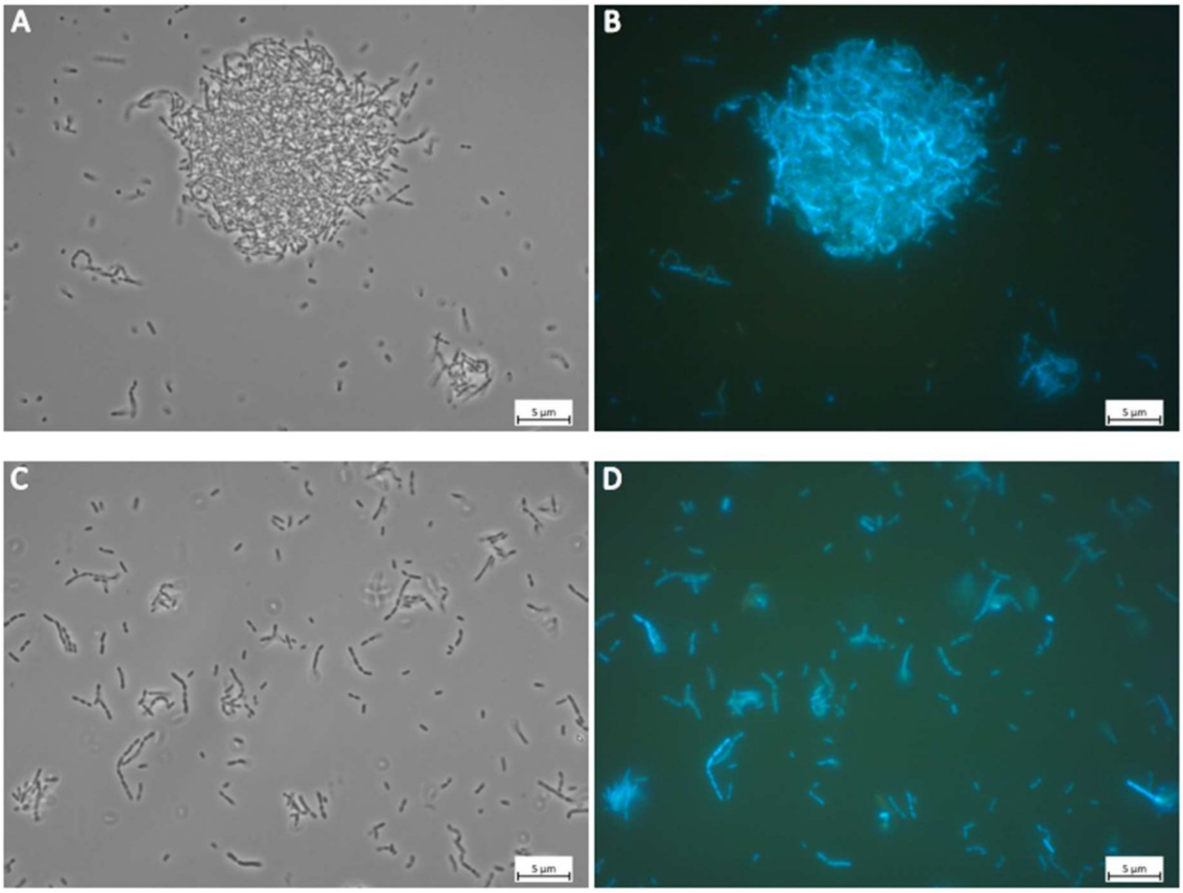

Supplementary Figure S6: Light- and corresponding fluorescence micrographs, displaying autofluorescence attributed to coenzyme F_{420} of the enriched archaeal cultures with co-enriched bacteria of the enrichments of A/B: P45 and C/D P86.

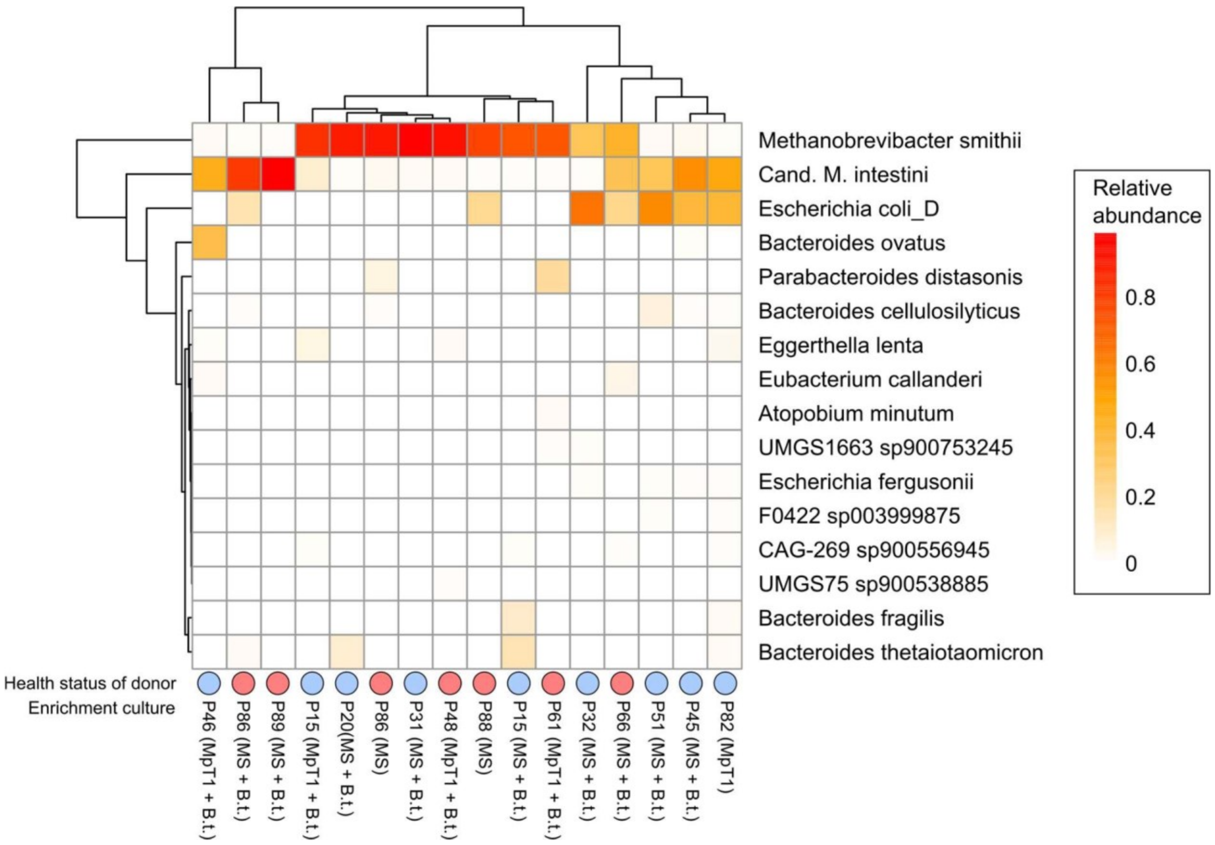

Supplementary Figure S7: Clustered heatmap of the relative abundances of archaeal and bacterial taxa in each enrichment culture. Shown are all bacterial taxa that were detected in a minimum of two archaeal enrichments. The color scale illustrates the percentage of the respective bacteria and archaea in the enrichment culture. For instance, enrichment culture P32 contained 64.341 % *Escherichia coli*_D, 1.189 %, *E. fergusonii*, 32.231 % *Methanobrevibacter smithii*, and 1.217 % Methanobrevibacter smithii_A. The colored dots indicate the health status of the donor for the respective enrichment culture (red: diseased, blue: healthy). Underlying data are available in the Source Data File and in our GitHub repository (Duller et al., 2024).

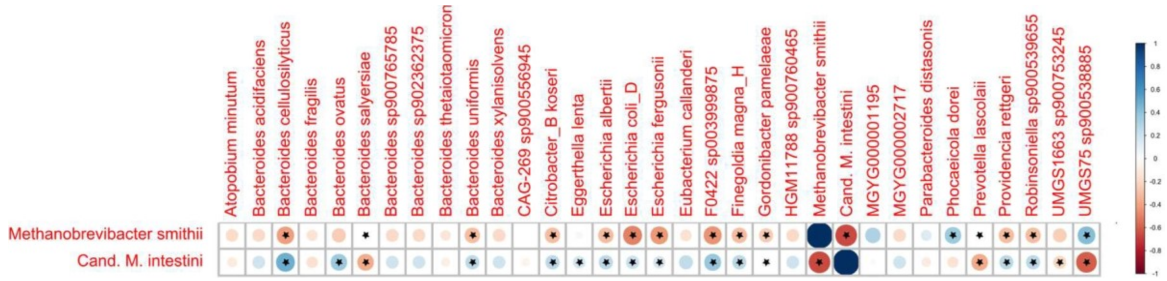

Supplementary Figure S8: Correlation matrix showing positive (blue dots) and negative correlations ($p < 0.05$ (two-sided), red dots; spearman correlation; Supplementary Data S4) **with co- enriched bacteria**. Statistically significant associations are highlighted by an asterisk. Underlying data are available in the Source Data File and in our GitHub repository (Duller et al., 2024).

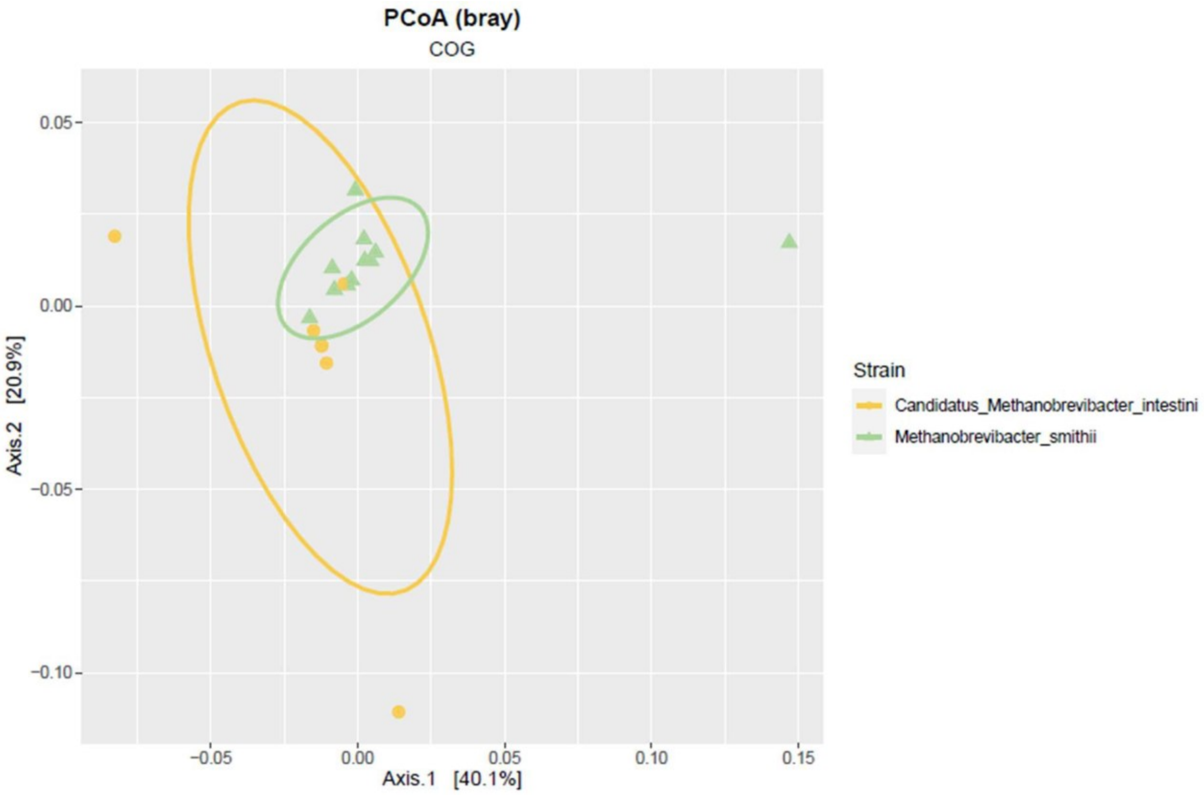

Supplementary Figure S9: PCoA, based on the presence/absence matrix obtained through functional annotation of the *M. smithii* (green) and *Cand.* M. intestini (yellow) isolates including reference genomes of *M. smithii* (Gut_genome132205) and *Cand.* M. intestini (Gut_genome143185)(Chibani et al., 2022). Underlying data are available in the Source Data File and in our GitHub repository (Duller et al., 2024).

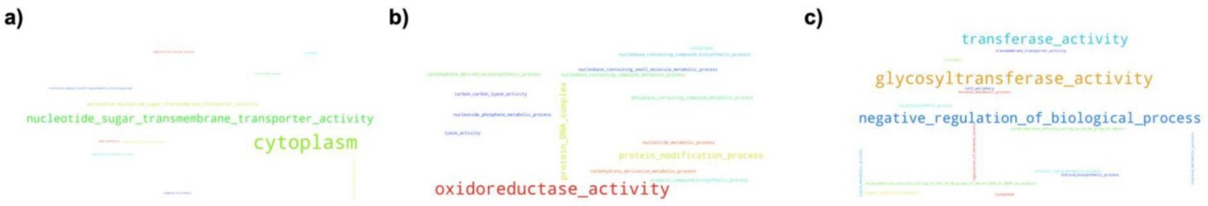

Supplementary Fig. S10. Occurrences of DeepFRI annotations (as Go term annotations) of otherwise unknown functions of *Cand.* M. intestini. a) 2AVB5, **b)** 2C5WB, **c)** 2DDW5. Only predictions with probability score ≥ 0.25 were considered.

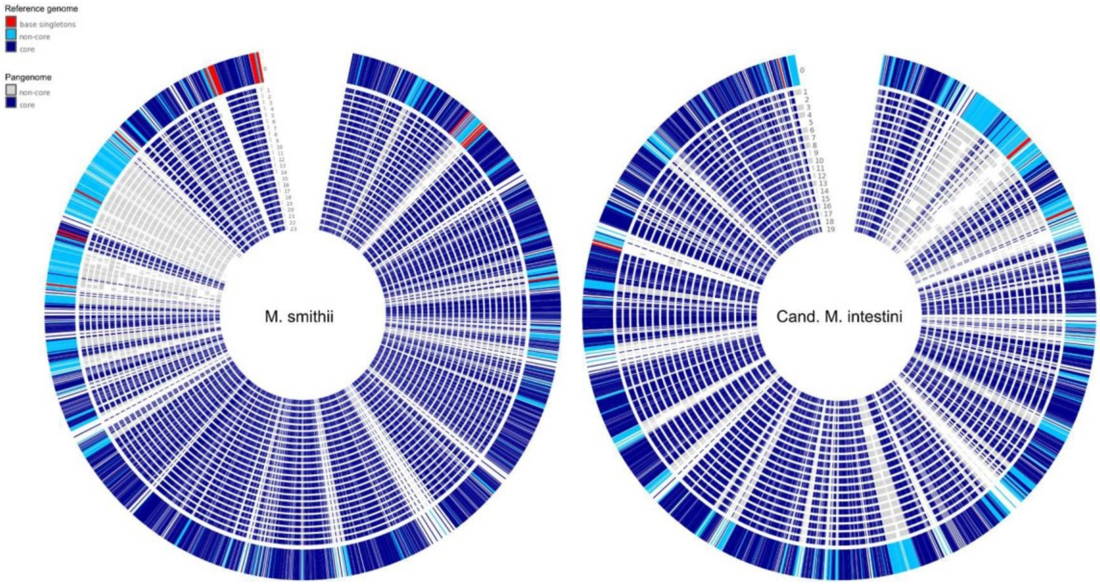

Supplementary Fig. S11: Pangenome circle plot of known *M. smithii* genomes (left) and *Cand.* M. intestini (right). Outer ring represents the representative genomes of *M. smithii* (Gut_genome132205;(Chibani et al., 2022) and *Cand.* M. intestini (Gut_genome143185; (Chibani et al., 2022). Only high-quality genomes and MAGs (> 90% completeness and < 10% contamination) from this and our previous study (Chibani et al., 2022) were included. Notably, the regions of non-core functions were fairly distributed across all genomes in *Cand*. M. smithii genomes, the *M. smithii* genomes revealed a more condensed area characterized of non-core functions.

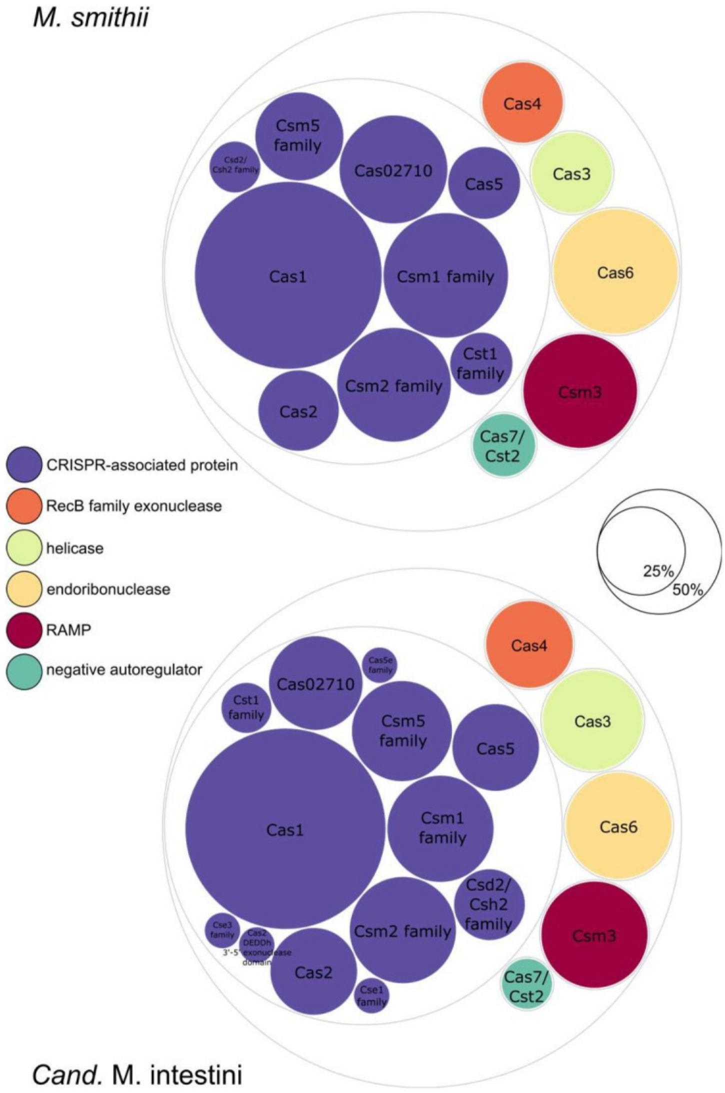

Supplementary Fig. S12: Inventory of CRISPR-associated genes in both species. Circle size indicates the presence in % of genomes. Analysis was based on pangenome analysis (*M. smithii* genomes: n=23; *Cand.* M. intestini genomes: n=19), with only high-quality genomes included. Underlying data are available in the Source Data File.

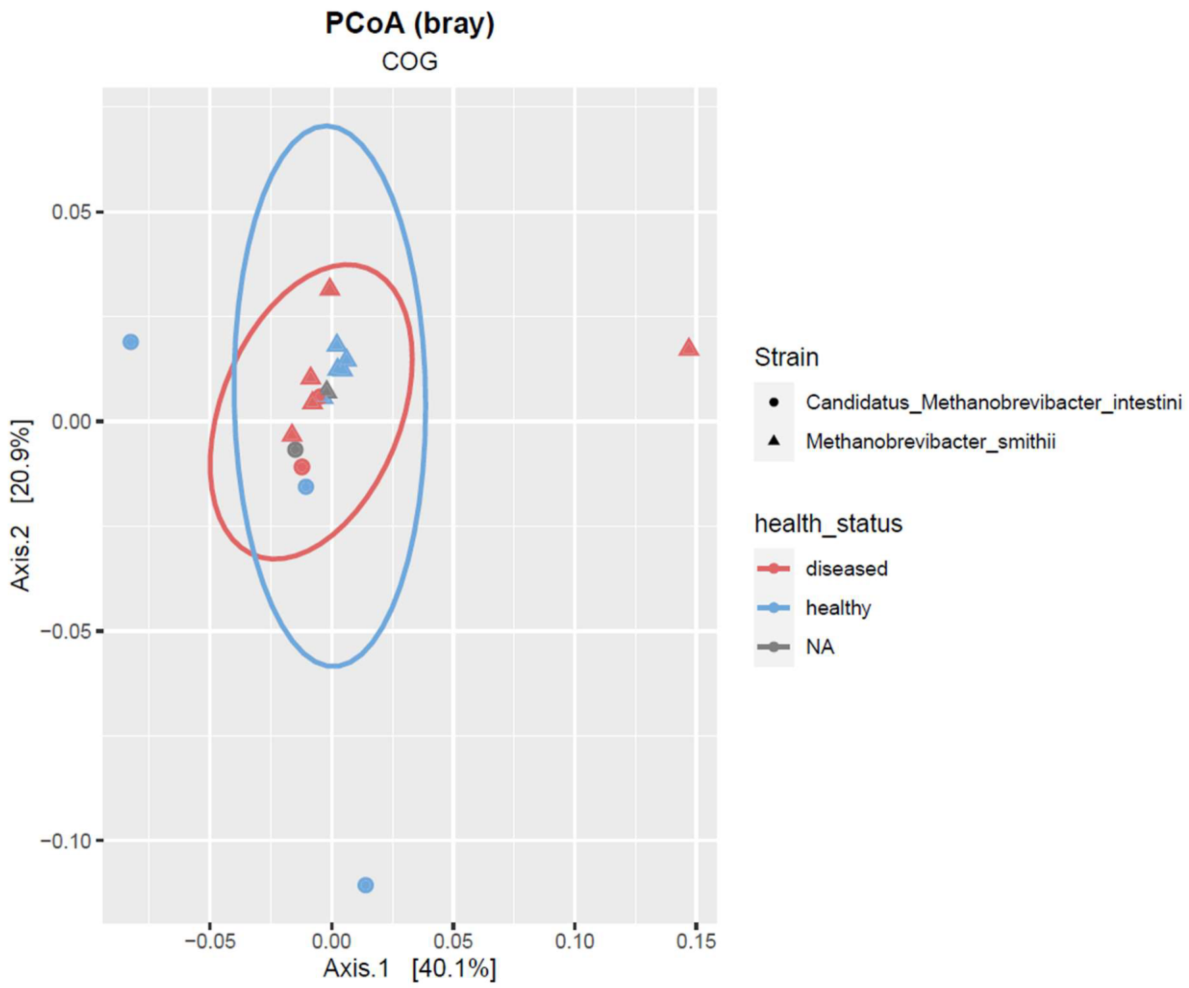

Supplementary Fig. S13: Comparison of the genomic inventory annotated through eggNOG with PcoA based on Bray-Curtis of the enriched *Methanobrevibacter* representatives (circle: *Cand.* M. intestini, triangle: *M. smithii*) of the diseased (red) and health (blue) study cohort. Reference genomes (Gut_genome132205, DSM2374 and Gut_genome143185; WWM1085; (Chibani et al., 2022)) could not be classified to a certain health status and are marked with NA (grey). Underlying data are available in the Source Data File.

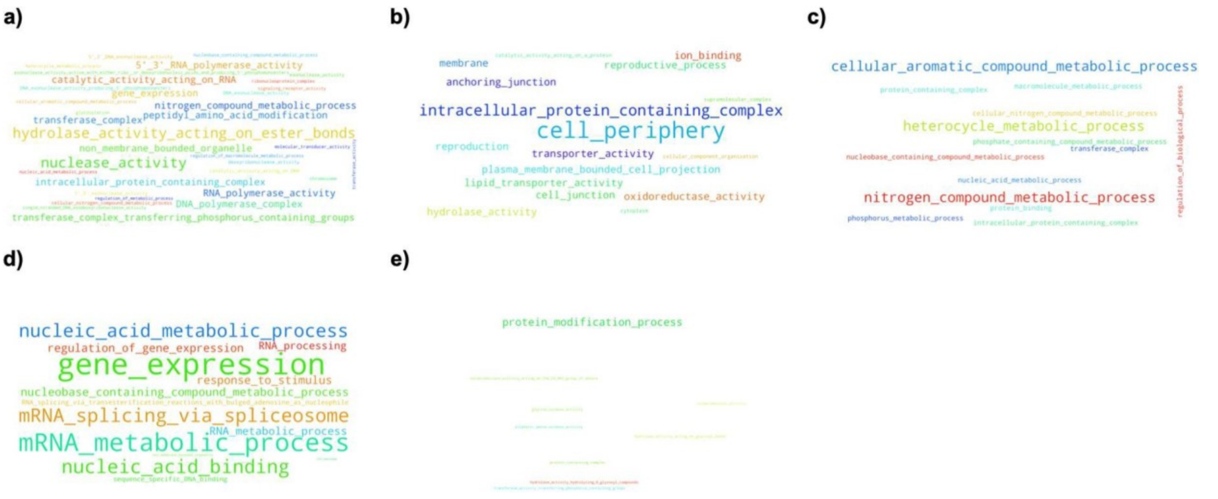

Supplementary Fig. S14: Occurrences of DeepFRI annotations (as GO term annotations) in diseased cohort **a)** arCOG5124, **b)** arCOG7602, **c)** COG3649, **d)** 2DP7E and **e)** 2DQUR. Only predictions with probability score ≥ 0.25 were considered. Underlying data are available in the Source Data File.

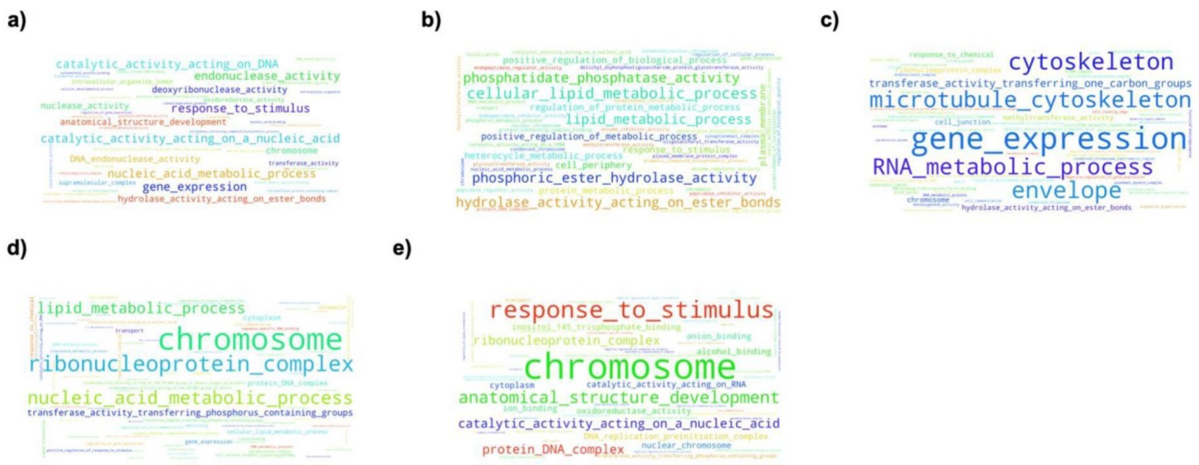

Supplementary Fig. S15: Occurrences of DeepFRI annotations (as GO term annotations) in healthy cohort **a)** COG0610, **b)** COG0670, **c)** COG4974, **d)** COG0286 and **e)** COG0732. Only predictions with probability score ≥ 0.25 were considered.

Underlying data are available in the Source Data File.

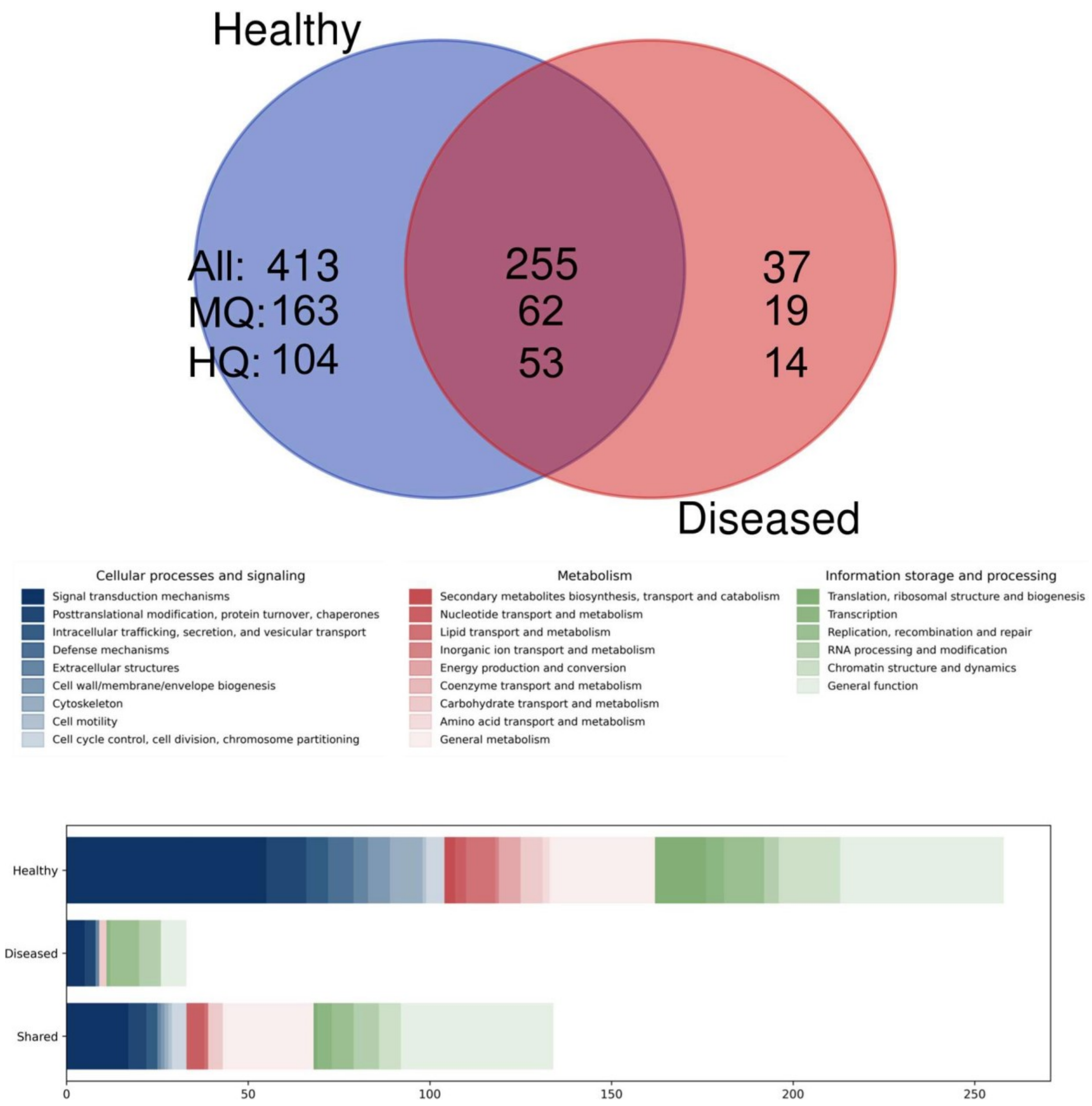

Supplementary Fig. S16. Comparison of Cand. M. intestini genomes from healthy and diseased cohorts, DeepFRI results. A) Venn diagram showing annotations of genes found exclusively in *Cand.* M. intestini genomes from healthy and diseased cohorts. MQ, medium quality (annotations with score ≥ 0.35); HQ, high quality (annotations with score ≥ 0.5). B) DeepFRI-derived annotations (MQ) as COG categories. Underlying data are available in the Source Data File.

REFERENCES

Chibani, C. M., Mahnert, A., Borrel, G., Almeida, A., Werner, A., Brugère, J.-F., Gribaldo, S., Finn, R. D., Schmitz, R. A., & Moissl-Eichinger, C. (2022). A catalogue of 1,167 genomes from the human gut archaeome. *Nature Microbiology*, *7*(1), 48–61. https://doi.org/10.1038/s41564-021-01020-9

Duller, S., Vrbancic, S., Szydłowski, Ł., Mahnert, A., Blohs, M., Predl, M., Kumpitsch, C., Zrim, V., Hoegenauer, C., Kosciolek, T., Schmitz, R. A., Eberhard, A., Dragovan, M., Schmidberger, L., Zurabischvili, T., Weinberger, V., Moser, A. M., Kolb, D., Pernitsch, D., … Moissl-Eichinger, C. (2024). *GitHub Repository Methanobrevibacter Enrichment*. DOI: 10.5281/Zenodo.13153860.
