# Supplementary material for: Targeted isolation of Methanobrevibacter strains from fecal samples expands the cultivated human archaeome
Source: Nat Commun. 2024 Aug 31;15:7593. doi: 10.1038/s41467-024-52037-7 (PMC11366006; doi:10.1038/s41467-024-52037-7)
Supplement: Supplementary file 3 — Description of Additional Supplementary Files [file 41467_2024_52037_MOESM3_ESM.docx]

**Description of Additional Supplementary Files**

File Name: Supplementary Data S1

Description: Overview of the 16 recruited participants and their metadata

File Name: Supplementary Data S2

Description: Overview of inclusion and exclusion criteria for participant recruitment

File Name: Supplementary Data S3

Description: Composition of enrichment cultures after antibiotics and lysozyme treatments

File Name: Supplementary Data S4

Description: Spearman correlations of co-cultured archaea and bacteria

File Name: Supplementary Data S5

Description: Recovered genomes, their classification according to GTDB, completeness and contamination.

File Name: Supplementary Data S6

Description: List of Methanobrevibacter genomes

File Name: Supplementary Data S7

Description: ANI Matrix

File Name: Supplementary Data S8

Description: ANI distances from different clusters to themselves and *Cand.* M. intestini (CMI, WWM1085) and *M. smithii* (MS, DSM2374).

File Name: Supplementary Data S9

Description: Kbase annotation output on CRISPR Functions for *M. smithii* and *Cand.* M. intestini

File Name: Supplementary Data S10

Description: Genomic and functional annotation of genes divergent in Methanobrevibacter genomes from diseased and healthy individuals
